# Post Ligation Cardiac Syndrome: an Educational Presentation

**DOI:** 10.21470/1678-9741-2020-0278

**Published:** 2022

**Authors:** Isaac Azevedo Silva, Ricardo Barros Corso, Glauco Pina, Marcus Vinicius Nascimento Santos, Helmgton José Brito de Souza, Maria Paula Meireles Fenelon, Leonardo Jadyr Silva Rodrigues Alves, Diane Lucio Vasconcelos, Viviane Bastos Paixão Marques

**Affiliations:** 1 Cardiovascular Associados - Cardiovascular Surgery Center, Brasília, Federal District, Brazil.; 2 Maternidade Brasília - Brasilia Maternity Hospital, Brasília, Federal District, Brazil.; 3 CEUB - Brasilia University Center, Brasília, Federal District, Brazil.

**Keywords:** Patent ductus arteriosus, Cardiovascular Surgical Procedures, Congenital heart disease

## Abstract

Although technically simple, surgical correction of patent ductus arteriosus can have serious complications. In this context, acute ventricular failure must be remembered, as its prompt diagnosis and proper management can change clinical outcomes.

**Table:** 

Abbreviations, Acronyms & Symbols	
Ao	= Aorta
CDA	= Closed ductus arteriosus
PA	= Pulmonary artery
PDA	= Patent ductus arteriosus
PLCS	= Post ligation cardiac syndrome

## INTRODUCTION

**Fig. 1:**
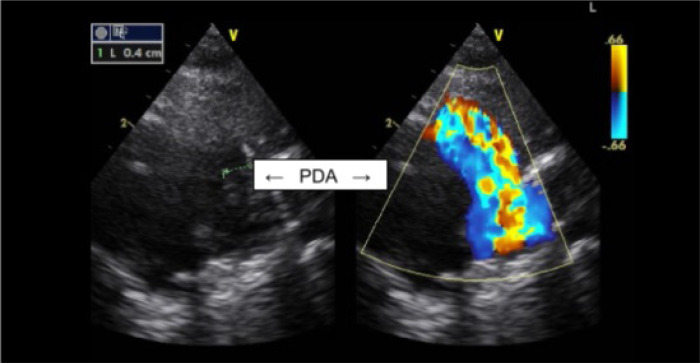
Transthoracic echocardiogram. PDA=patent ductus arteriosus.

**Fig. 2:**
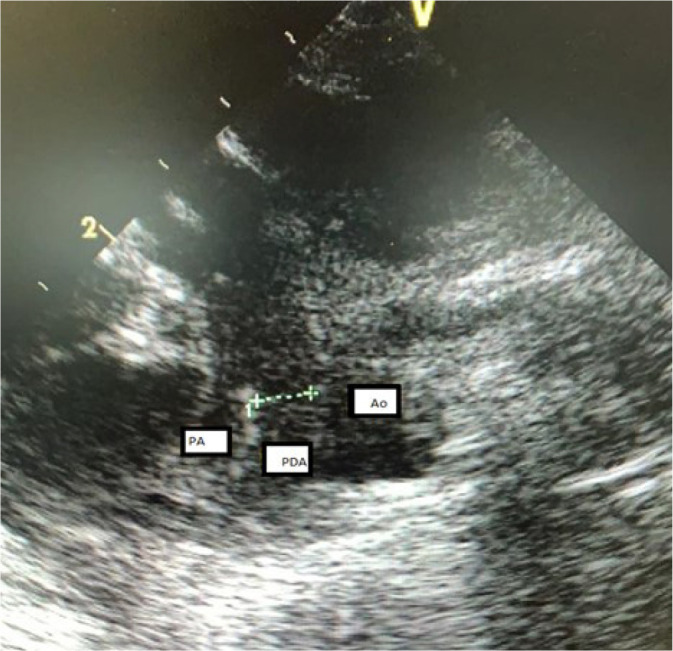
Transthoracic echocardiogram. Ao=aorta; PA=pulmonary artery; PDA=patent ductus arteriosus.

The patient was newborn, female, preterm (gestational age: 29 weeks), 14 days old, and diagnosed with patent ductus arteriosus (PDA), measuring 4.0 mm in diameter, with hemodynamic repercussion, refractory to attempted drug closure (Figures 1 and 2). She evolved with worsening of ventilatory parameters and a huge splanchnic hypoperfusion. Therefore, urgent surgical ligation of the PDA was performed through a left minithoracotomy, using metal clips. The procedure was performed uneventfully. However, eight hours after surgery, the patient faced severe hypotension and hypoxemia, requiring high mechanical ventilation parameters.

## QUESTIONS

A. What is the cause of this clinical worsening?

B. How this diagnose (Question A) can be confirmed?

C. Explain its pathophysiologic patterns.

D. Describe the best approach for this condition

### Discussion of Questions:

**Question A.** Clinical deterioration with severe hypotension initiating after few hours of a surgery for PDA correction is compatible with cardiogenic shock, a condition known as post ligation cardiac syndrome (PLCS).

**Question B.** Transthoracic echocardiogram is a fast and accurate method for confirming the diagnose of PLCS. It is usually available and can be performed at bedside in the neonate intensive care unit.

**Fig. 3:**
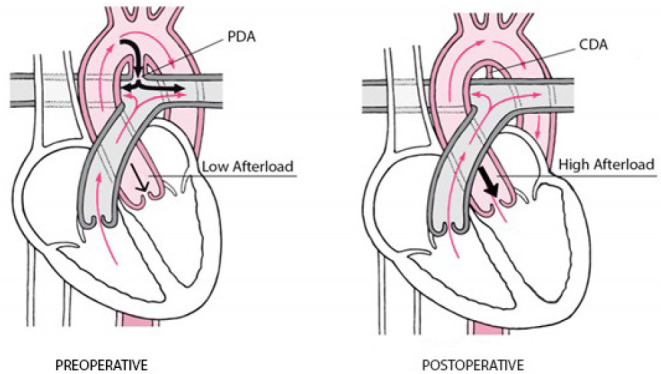
Preoperative condition (low afterload) and postoperative result (high afterload). CDA=closed ductus arteriosus; PDA=patent ductus arteriosus.

**Question C.** PLCS is related to an acute increase in afterload and a decrease in preload (Figure 3) due to closure of the ductus arteriosus. In a PDA condition, the pulmonary vascular bed offers low resistance to the left ventricle (low afterload), and, in consequence of high pulmonary flow, the left atrium is overloaded (high preload). However, as soon as the ductus was ligated, the left ventricle faced an acute elevation in afterload (no more low resistant pulmonary vascular bed) and a reduction in preload. So, systolic and diastolic dysfunction may occur, leading to a reduction in cardiac output. Clinically, the patient shows systemic arterial hypotension, oxygenation lability, need for vasoactive drugs, and worsening respiratory function ^[1-3]^.

**Question D.** The approach to PLCS should be based on afterload reduction and inotropic support using dobutamine or milrinone, and, in addition, volume expansion may be established to increase preload^[^1^]^. Vasopressors like epinephrine may be used, if strictly necessary and in the lowest effective dose, in order to not increase substantially the afterload, which could impair heart function.

## BRIEF CONSIDERATION OF THE CASE REPORTED

Due to the progressive clinical deterioration after an ordinary operating room status, an urgent echocardiogram was performed, showing an important left ventricular dysfunction. So, PLCS was considered, and the treatment was promptly instituted with infusion of dobutamine, low dose epinephrine, and careful fluid management. There was progressive improvement until full ventricular recovery and weaning from inotrope five days later and from mechanical ventilation on the ninth day. The patient was discharged healthy.

## LEARNING POINTS

- PDA is one of the most common congenital heart defects, accounting for 5%-10% of all congenital heart diseases^[4]^.
- Treatment options include conservative, pharmacological, and surgical approaches^[3]^.
- PLCS is a rare but serious complication characterized by cardiovascular and pulmonary maladaptation after surgical correction of PDA, resulting in a severe low cardiac output status^[1]^.
- This condition is life-threatening and a proper afterload and preload control is mandatory^[1]^.

**Table:** 

Authors’ Roles & Responsibilities	
IAS	Substantial contributions to the design of the work; and the acquisition and analysis of data for the work; drafting the work; final approval of the version to be published
RBC	Substantial contributions to the design of the work; and the acquisition and analysis of data for the work; drafting the work; final approval of the version to be published
GP	Substantial contributions to the design of the work; and the acquisition and analysis of data for the work; drafting the work; final approval of the version to be published
MVNS	Substantial contributions to the design of the work; and the acquisition and analysis of data for the work; drafting the work; final approval of the version to be published
HJBS	Substantial contributions to the design of the work; and the acquisition and analysis of data for the work; drafting the work; final approval of the version to be published
MPMF	Substantial contributions to the design of the work; and the acquisition and analysis of data for the work; drafting the work; final approval of the version to be published
LJSRA	Substantial contributions to the design of the work; and the acquisition and analysis of data for the work; drafting the work; final approval of the version to be published
DLV	Substantial contributions to the design of the work; and the acquisition and analysis of data for the work; drafting the work; final approval of the version to be published
VBPM	Substantial contributions to the design of the work; and the acquisition and analysis of data for the work; drafting the work; final approval of the version to be published
